# An Analysis of Behaviour Change Techniques Used in a Sample of Gestational Weight Management Trials

**DOI:** 10.1155/2016/1085916

**Published:** 2016-02-29

**Authors:** H. Soltani, M. A. Arden, A. M. S. Duxbury, F. J. Fair

**Affiliations:** ^1^Centre for Health and Social Care Research, Sheffield Hallam University, Montgomery House, 32 Collegiate Crescent, Sheffield S10 2BP, UK; ^2^Department of Psychology, Sociology & Politics, Sheffield Hallam University, Heart of the Campus, Collegiate Crescent, Sheffield S10 2BQ, UK

## Abstract

*Introduction*. Maternal obesity and excessive gestational weight gain are associated with multiple adverse outcomes. There is a lack of clarity on the specific components of effective interventions to support pregnant women with gestational weight management.* Method*. All 44 studies within a preexisting review of lifestyle interventions, with a potential to impact on maternal weight outcomes, were considered for content analysis. Interventions were classified using Behaviour Change Technique (BCT) taxonomy clusters to explore which categories of BCT were used in interventions and their effectiveness in managing gestational weight gain.* Results*. The most commonly used BCTs were within the categories of “feedback and monitoring,” “shaping knowledge,” “goals and planning,” “repetition and substitution,” “antecedents,” and “comparison of behaviours.” For diet and mixed interventions “feedback and monitoring,” “shaping knowledge,” and “goals and planning” appeared the most successful BCT categories.* Conclusions*. Poor reporting within studies in defining the BCTs used, in clarifying the differences in processes between intervention and control groups, and in differentiating between the intervention and research processes made BCT classification difficult. Future studies should elaborate more clearly on the behaviour change techniques used and report them accurately to allow a better understanding of the effective ingredients for lifestyle interventions during pregnancy.

## 1. Introduction

Maternal obesity and excessive gestational weight gain are associated with adverse outcomes (such as macrosomia, shoulder dystocia, and gestational diabetes [1, 2]) and are on the rise. Despite an urgent need for evidence based guidance to support pregnant women on gestational weight management, there is a lack of clarity about effective interventions and their specific components. Interventions developed to reduce excessive gestational weight gain and its associated outcomes generally fit into the broad categories of dietary only, physical activity only, and mixed approaches utilising both diet and physical activity components [3]. It is important to identify which components and specific behaviour change techniques within these complex interventions are most effective, since this is needed to inform the development of future interventions and guidance.

Michie et al. have reported a consensually agreed structured taxonomy of behaviour change techniques which provides a framework for a more precise reporting of complex interventions [4]. The Behaviour Change Technique (BCT) taxonomy [4] is a useful tool to extract the active components of interventions, allowing comparisons between the component parts of successful and unsuccessful behaviour change interventions. Several studies [5–7] have used the behaviour change technique taxonomy described by Michie et al. [8] to define gestational weight gain management interventions. However only Currie et al. [9] have used the most up-to-date clustered BCT taxonomy [4] to code lifestyle interventions during pregnancy or the postnatal period, in their systematic review of 14 studies aimed at reducing the decline in physical activity during pregnancy.

Gestational weight management strategies often rely on complex interventions involving various interacting components. Identification of active components of these interventions would help in better understanding and interpreting the results of the existing systematic reviews. It would also be helpful to inform the design of new interventions and their evaluations.

Numerous systematic reviews have evaluated the efficacy of interventions designed to improve weight outcomes for mothers [3, 5, 6, 10–14]. Of these most included 9 to 11 interventional studies [5, 10, 11, 13], with one review [14] only including 5 studies, two reviews including 19 studies [6, 12], and the final review by Thangaratinam et al. [3] of 44 studies. The reviews by Brown et al. [14], Thangaratinam et al. [3], and Choi et al. [13] focused exclusively on randomised controlled trials. Results across the reviews have varied. Streuling et al. [10] found that physical activity or diet alone interventions were not effective at reducing gestational weight gain but interventions based on physical activity and dietary counselling combined with weight monitoring appeared to be successful. In comparison Choi et al. [13] found that obese and overweight women allocated to physical activity or physical activity plus diet interventions in pregnancy had lower gestational weight gains, with supervised physical activity being especially effective. Thangaratinam et al. [3] showed some evidence of effectiveness across all interventions in reducing gestational weight gain (mean difference (MD) −1.42, 95% confidence interval (CI) −1.89 to −0.95). They also reported significant reductions in weight gain in pregnancy in subgroup analysis for dietary interventions (MD −3.84, 95% CI −5.22 to −2.45), physical activity interventions (MD −0.72, 95% CI −1.20 to −0.25), and interventions with a mixed approach (MD −1.06, 95% CI −1.67 to −0.46).

Due to the comprehensive approach in inclusivity and rigour in Thangaratinam et al.'s [3] review and due to it being the most highly accessed and cited article within the field of research of gestational weight management, this was selected as the source of literature for content analysis in our review. The aim of this study was therefore to evaluate the behaviour change techniques included in diet, physical activity, or mixed interventions with a potential to impact on maternal or fetal outcomes related to weight and to identify the categories of behaviour change technique of those interventions which were effective. To our knowledge, this is the first study to use the BCT taxonomy to identify techniques used in a wide range of gestational weight management lifestyle interventions.

### 1.1. Objectives

To explore the patterns of behaviour change techniques used in interventions with a potential to impact maternal and fetal outcomes related to gestational weight gain.

## 2. Methods

### 2.1. Data Selection

This study was based on the 44 randomised controlled trials of interventions with a potential to impact maternal or fetal outcomes related to weight which were included in the HTA commissioned systematic review [3]. The studies included in the review were focused on diet only (*n* = 13), physical activity only (*n* = 18), or mixed (*n* = 13) diet and physical activity interventions for a range of pregnant women, focussing specifically on overweight and or obese women in 11 studies. The study selection criteria and assessments of quality and bias have all been reported by Thangaratinam et al. [3]. They found that the quality of studies included in the analysis for gestational weight gain was moderate, but quality for other outcomes such as preterm delivery and hypertension was low, where there may have been a risk of publication bias.

### 2.2. Data Extraction and Synthesis

Michie et al.'s [4] health behaviour change technique taxonomy was used to identify the behavioural components of the intervention within each included study. This taxonomy contains 93 itemised health behaviour change techniques which are clustered into 16 groupings (see the following list), with each group containing between 3 and 11 clustered behaviour change techniques. For practicality of reporting the category groupings were used for the purpose of this review.

*Groupings within Michie et al.'s [4] Hierarchically Clustered Behaviour Change Technique Taxonomy. *Consider the following:

1. Goals and planning.
2. Feedback and monitoring.
3. Social support.
4. Shaping knowledge.
5. Natural consequences.
6. Comparison of behaviour.
7. Associations.
8. Repetition and substitution.
9. Comparison of outcomes.
10. Reward and threat.
11. Regulation.
12. Antecedents.
13. Identity.
14. Scheduled consequences.
15. Self-belief.
16. Covert learning.

Three researchers (H. Soltani, M. A. Arden, and A. M. S. Duxbury) independently extracted and coded the data, to improve reliability of the data categorisation. Where there were differences in coding, the research team had a discussion to reach consensus regarding the codes and categories.

Behaviour change technique categories were classified as successful or unsuccessful within each study dependent upon whether a significant difference was found between the intervention and control group on gestational weight gain. Due to the heterogeneity of the included studies data was synthesised narratively and presented in tables and graphs as statistical synthesis was not possible.

## 3. Results

Of the original 44 papers included within the Thangaratinam et al. review [3], one study only consisted of a conference abstract [26]. Full-text versions of all of the other articles were obtained. The 44 trials included 7627 women who had been randomised. Healthcare professionals delivering the interventions varied across the studies and included dieticians, nutritionists, clinical psychologists, doctor, nurses, and midwives.

**Figure 1:**
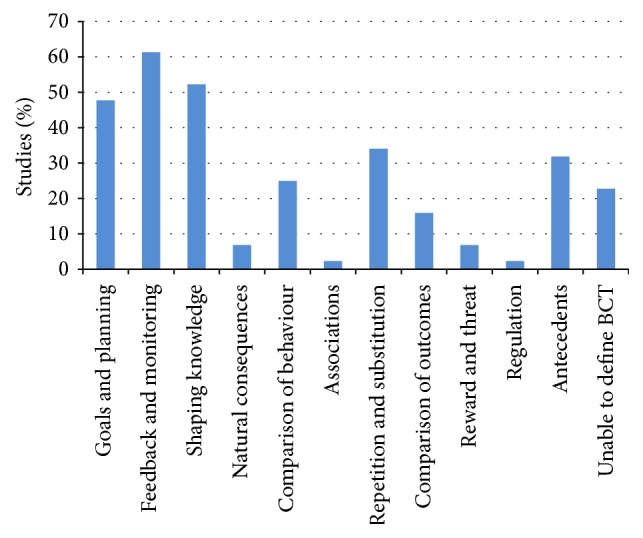
Behaviour change technique taxonomy categories of the interventions in included studies (*n* = 44 studies).

Table 1 contains study characteristics and the behaviour change technique categories agreed by the researchers for each of the included studies [15–59]. It was not possible to apply any behaviour change taxonomy code to 10 of the studies.  Figure 1 shows the distribution of BCT categories within the studies. The most commonly used behaviour change technique clusters were “feedback and monitoring,” “shaping knowledge,” “goals and planning,” “repetition and substitution,” “antecedents,” and “comparison of behaviours.”

There were many studies where the authors could not agree on the behaviour change techniques involved within the intervention. The disputed techniques are shown in Table 2. Eight of the 10 studies for which no behaviour change technique code was recorded had potentially included BCTs but the research team could not reach agreement on them. Two studies [27, 56] included no discernible BCTs. The most common category where a disagreement occurred between the authors was “goals and planning,” with 21 of the 22 studies with a disputed behaviour change technique being discrepant within this cluster. In only 2 of these 21 studies [21, 23] was the discrepancy not within the subcategory “goal setting (behaviour).”

**Figure 2:**
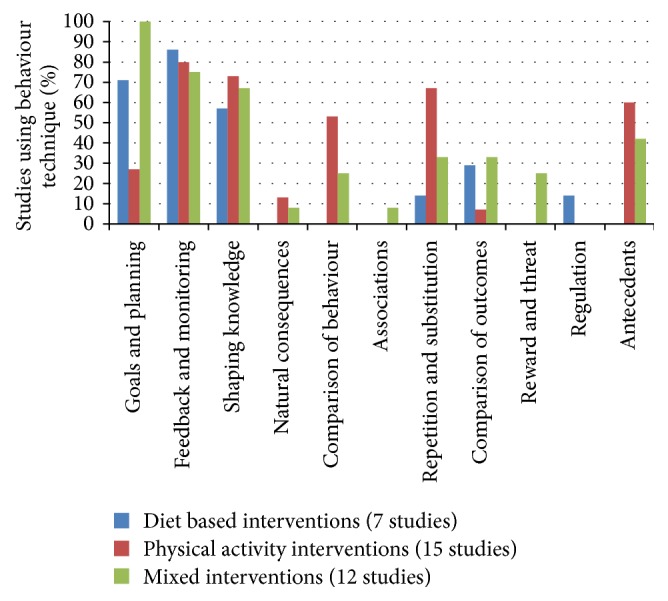
Behaviour change technique taxonomy categories according to intervention type (*n* = 34 studies).

For the studies where it was possible to categorise the type of behaviour change, BCT category according to type of intervention was plotted (Figure 2). While all types of intervention made use of “feedback and monitoring” and “shaping knowledge” techniques physical activity based interventions utilised “comparison of behaviours” and “repetition and substitution” more than dietary or mixed lifestyle interventions. In comparison dietary based and mixed interventions incorporated “goals and planning” more often.

**Figure 3:**
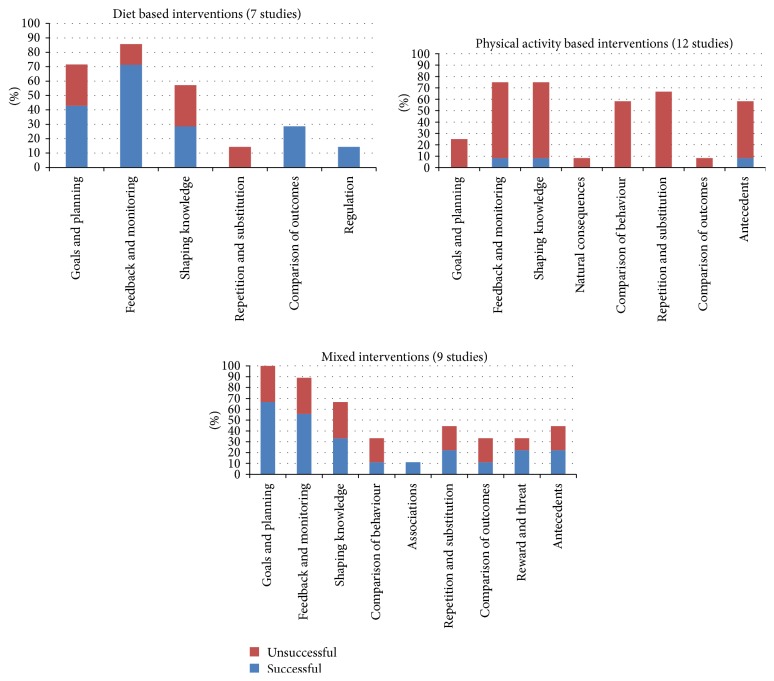
Success of intervention on gestational weight gain across intervention type.

Gestational weight gain was reported in 34 studies; however for 6 of these studies no agreement was obtained for applying a BCT code. The success of each behaviour change technique according to type of intervention in the resulting 28 studies is shown in Figure 3. In studies where a BCT classification could be applied a significant difference in gestational weight gain between the intervention groups and control groups was found more often for diet based (*n* = 5) or mixed interventions (*n* = 6) compared to physical activity based interventions (*n* = 1).

The prevalence of each BCT category in both successful and unsuccessful interventions for reducing gestational weight gain is shown in Table 3. The BCT categories present in 50% or over of the studies with successful interventions were “feedback and monitoring,” “goals and planning,” and “shaping knowledge.”

## 4. Discussion

We have used the Thangaratinam et al. [3] review as an example of a report incorporating diet, physical activity, and mixed lifestyle interventions with the potential to impact on maternal or fetal weight outcomes. Of the 44 studies included within that review, 34 reported total gestational weight gain.

The most commonly used behaviour change technique categories were “feedback and monitoring,” “shaping knowledge,” “goals and planning,” “repetition and substitution,” “antecedents,” and “comparison of behaviours.” To our knowledge there is only one other study [9] in which lifestyle interventions in pregnancy or the postpartum have been classified according to Michie et al.'s BCT taxonomy [4]. The behaviour change technique components of interventions in pregnancy aimed at reducing the decline in physical activity were categorised within that study by Currie et al. [9], with the 6 most commonly used BCT categories being the same as those found within this study. Others have used Michie's previous taxonomy [8] to code pregnancy and postpartum lifestyle interventions. All of these found behaviour change techniques within the categories of “goals and planning” and “feedback and monitoring” were the most frequently used [5–7]. Hill et al. [6] and Gilinsky et al. [7] both also noted “instruction on how to perform the behavior” was often utilised which sits within the “shaping knowledge” cluster in the Michie et al. BCT taxonomy [4]. Gilinsky et al. [7] also identified “set graded tasks” which is often used in physical activity trials and is classified under the “repetition and substitution” cluster. Hill et al. [6] found studies often provided “information on the consequences of behavior” which corresponds with behaviours in the “natural consequences” cluster. With the exception of Hill et al.'s [6] “natural consequences” category, these behaviour change techniques correspond closely with those found in our study.

When assessing BCT taxonomy categories, there were disputes among the authors (Table 2), mostly around the “goal setting (behaviour)” technique. This categorisation was disagreed on for 15 out of the 18 physical activity interventional studies which could account for “goals and planning” appearing to be more often incorporated into dietary based and mixed interventions compared to physical activity interventions. In the majority of these disputed studies there was no explicit reference to goal setting within the descriptions of the intervention procedures provided according to the BCT taxonomy definition: “set or agree on a goal defined in terms of the behaviour to be achieved” [4]. Participants had been assigned to the intervention condition as part of the research protocol. Although the intervention description included exercise classes or similar, it was not clear whether or not a goal had been set or agreed to attend/engage in these classes, even though this seemed likely to have occurred. These disagreements may reflect health psychologists stricter understanding and interpretation of BCT coding, which does not necessarily match the understanding of clinicians and emphasises the potential difficulties of translating BCT's into practice. Clarification of these ambiguities is required to enhance the implementation and reporting of BCT's in research and practice.

Categories of behaviour change techniques were present in both effective and ineffective interventions, except for “regulation” which was only present in one successful diet based intervention and “association” which was within one successful mixed intervention. Others who have assessed behaviour change techniques utilised within interventions have similarly found behaviour change strategies to be present in both effective and ineffective studies [5]. Within this current study physical activity interventions were largely unsuccessful at managing gestational weight gain, whereas individual behaviour change techniques within diet based or mixed interventions were of varied success. However the success or failure of an intervention could be a result of a number of factors beyond the specific BCT's, for example, the study design, insufficiency of the sample size, or poor fidelity to intervention processes and attrition rates in the original studies.

The success or failure of the interventions may have been influenced by individual BCTs or by the specific combination of BCTs within the intervention. It was not possible to statistically analyse the individual effectiveness of BCTs or to assess the effectiveness of different combinations of behaviour techniques due to the number of different combinations of BCTs present within studies, which is a limitation of this review. However it was noted that successful interventions always included BCTs from one or both of “goals and planning” or “monitoring and feedback”. This is in line with Michie et al.'s [60] findings with regard to healthy eating and physical activity interventions in the general population, with what Gilinsky et al. [7] found for interventions effective at increasing postnatal physical activity and with Harkin et al. [61] who found larger effect sizes in interventions incorporating monitoring of goal progress. When specifically looking at gestational weight gain studies utilising explicit goal setting Brown et al. [14] found a difference in the types of interventions which were effective at different body mass indexes (BMIs) with some interventions working best for women of normal weight and others for women who were overweight or obese. Future research into effective behaviour change techniques will need to take account of potential differential effects across various BMI categories.

The lack of clear and consistent reporting of which behaviour change techniques were undertaken within each intervention was a recurrent theme across this study. Poor reporting, making classification of BCTs difficult, was noted to occur within three main areas: lack of differentiation between the intervention processes and the research processes of the study; difficulties in determining which components were delivered only to the intervention group rather than to both the intervention and control groups; and finally poor or vague definitions of the behaviour change components used. Each of these areas will be discussed in turn.

Some studies were noted to lack clarity over whether the incorporated behaviours were part of the intervention or just part of the study design, for example, glucose monitoring, blood pressure measurements, and completing questionnaires. If these activities were purely for the researchers own benefit to determine clinical outcome measures for the study they would not be part of the intervention and therefore should not be part of the behaviour change technique classification; however if participants were given feedback on the results of blood pressure readings or their current weight in order to promote behaviour change then these procedures would be part of the intervention and their component techniques should be classified This lack of clarity across the studies made BCT classification difficult. The importance of clear reporting was also highlighted due to difficulties in determining which behavioural processes were solely applied to the intervention group. For example, statements such as “participants were weighed at each appointment” did not make it clear if everyone was weighed or just the intervention group.

Behaviour change technique coding was difficult as some studies used vague phrases such as “nutrition counselling” or “education” to describe their interventions and did not clearly specify what techniques these interventions included. Furthermore interventions such as water aerobics sessions or gym access where a fitness instructor was present would most likely include “how to perform the behavior” or “demonstrating the behaviour”; however when this was not explicitly stated it was difficult to identify the techniques and their effectiveness in a standardised and consistent manner. Others have also described the difficultly of applying behaviour change codes to intervention components due to a lack of specificity within reports [5].

One study noted by the authors to provide a clear description which allowed rigorous behaviour change technique codes to be applied was Jeffries et al. [49]. Codes included “goal setting the outcome” as intervention women were informed of their optimal weight gain based on their BMI and Institute of Medicine (IOM) guidelines and given personalised weight gain charts and “self-monitoring the outcome” as intervention group women were asked to weigh themselves every 4 weeks and record it on their chart. In contrast an example of reporting which made BCT classification difficult is Bechtel-Blackwell et al. [22]. They conducted an education based intervention where the intervention group had three 20 minute group sessions which covered:* “nutritional needs specific to the woman's stage of her pregnancy.” *It was not clear whether these sessions just provided information or worked through problems to provide solutions (i.e., if you feel sick, then drink water or go for a walk). No code could therefore be applied.

When developing intervention studies researchers should “clearly define and provide a rationale for all behaviour change techniques that have been included” [62]. Future studies should use frameworks for intervention design such as the Behaviour Change Wheel [63] that guide developers through the process of developing a clear rationale based on evidence. Reporting behaviour change interventions stating what has been done using the standardised terms found in the behaviour taxonomy would enable other researchers to understand exactly what the intervention included and would allow statistical analysis to evaluate the effectiveness of specific study components. This would provide a more robust conclusion of the effectiveness of specific BCT categories at preventing excessive gestational weight gain, facilitating the replication of successful interventions or intervention components. The lack of standardised terms in the maternal obesity intervention literature, and the use of vague terms such as “nutrition counselling” means that we cannot understand what aspects of the intervention made it successful and that we cannot properly replicate it in future research. Without the ability to build on knowledge in this way researchers will not be able to improve intervention design in the future.

## 5. Conclusions

Coding interventions using the BCT taxonomy is valuable in the field of gestational weight management. However a better understanding of these techniques, clarity in their implementation, and reporting in a standard format are necessary to allow a robust and reliable evaluation of their efficacy.

## Figures and Tables

**Table 1:** Study characteristics and definite Behaviour Change Technique categories.

Study	Intervention delivery	Number of participants randomised	Number of participants analysed	GWG in kg		Significant difference	Agreed BCT categories
				Intervention group mean (SD)	Control group mean (SD)		
Diet based interventions							
Clapp 1997 [15]	Not stated	—	12	11.8 (5.6)	19.7 (2.9)	*p* < 0.01	—
Crowther et al. 2005 [16]	Dietician	1000	1000	8.1 (0.3)	9.8 (0.4)	Adjusted *p* = 0.01	Feedback and monitoring, shaping knowledge, comparison of outcomes
Landon et al. 2009 [17]	Clinician	958	931	2.8 (4.5)	5 (3.3)	*p* < 0.001	Feedback and monitoring
Ney et al. 1982 [18]	Not stated	20	20	11.8 (4.5)	15.9 (6.8)	*p* < 0.05	—
Quinlivan et al. 2011 [19]	Food technologist, clinical psychologist	132	124	7.0 (5.2)	13.8 (5.2)	*p* < 0.001	Goals and planning, feedback and monitoring, shaping knowledge, regulation
Thornton et al. 2009 [20]	Dietician, physician	257	232	4.99 (6.79)	14.06 (7.40)	*p* < 0.001	Goals and planning, feedback and monitoring
Wolff et al. 2008 [21]	Dietician	64	50	6.6 (5.5)	13.3 (7.5)	*p* = 0.002	Goals and planning, feedback and monitoring, comparison of outcomes
Bechtel-Blackwell 2002 [22]	Research nurse	60	46	6.87 (NR)	5.57 (NR)	NS	—
Briley et al. 2002 [23]	Nutritionist	27	20	11.9 (6.3)	15.2 (5.1)	NS	Goals and planning, feedback and monitoring, shaping knowledge
Khoury et al. 2005 [24]	Dietician	290	290	8.9 (3.1)	9.4 (3.0)	NS *p* = 0.20	Goals and planning, shaping knowledge, repetition and substitution
Rae et al. 2000 [25]	Research dietician	125	117	11.56 (10.48)	9.68 (10.66)	NS *p* = 0.338	—
Badrawi et al. 1992 [26]	Not stated	100	—	NR	NR	NR	—
Gomez et al. 1994 [27]	Not stated	60		NR	NR	NR	—

Physical activity based interventions							
Sedaghati et al. 2007 [28]	Qualified instructor and midwife	100	90	13.55 (1.131)	15.1 (2.102)	*p* < 0.000	Feedback and monitoring, shaping knowledge, antecedents
Baciuk et al. 2008 [29] Cavalcante et al. 2009 [30]	Qualified instructor	71	70	14.3 (2.1)	15.1 (1.6)	NS *p* = 0.38	Feedback and monitoring, shaping knowledge, comparison of behaviour, repetition and substitution, antecedents
Barakat et al. 2009 [31]	Qualified fitness specialist	160	142	11.5 (3.7)	12.4 (3.4)	NS (but some difference in obese only group)	Feedback and monitoring, shaping knowledge, comparison of behaviour, repetition and substitution, antecedents
Barakat et al. 2012 [32]	Qualified instructor with obstetric assistance	100	83	12.5 (3.2)	13.8 (3.1)	NS *p* > 0.05	Feedback and monitoring, shaping knowledge, comparison of behaviour, repetition and substitution, antecedents
Clapp et al. 2000 [33]	Not stated	50	46	15.7 (4.7)	16.3 (3.4)	NS	—
Erkkola 1976 [34]	Not stated	76	62	11.8 (NR)	11 (NR)	NS	Feedback and monitoring, shaping knowledge, natural consequences
Garshasbi and Faghih 2005 [35]	Midwife	266	212	14.1 (3.8)	13.8 (5.2)	NS *p* = 0.63	Comparison of outcomes
Haakstad and Bo 2011 [36]	Qualified instructor	105	105	13 (4)	13.8 (3.8)	NS *p* = 0.31	Goals and planning, feedback and monitoring, shaping knowledge, comparison of behaviour, repetition and substitution
Hopkins et al. 2010 [37]	Not stated	98	84	8.2 (3.5)	8 (3.7)	NS	Goals and planning, feedback and monitoring, antecedents
Marquez-Sterling et al. 2000 [38]	Qualified instructor	20	15	16.2 (3.4)	15.7 (4)	NS *p* = 0.649	Goals and planning, comparison of behaviour, repetition and substitution
Ong et al. 2009 [39]	Supervised	12	12	3.7 (3.4)	5.2 (1.3)	NS *p* = 0.155	Shaping knowledge, repetition and substitution, antecedents
Prevedel et al. 2003 [40]	Not stated	60	41	15 (NR)	12.7 (NR)	NS	Feedback and monitoring, shaping knowledge, comparison of behaviour, repetition and substitution
Santos et al. 2005 [41]	Not stated	92	72	5.7 (NR)	6.3 (NR)	NS *p* = 0.62	Feedback and monitoring, shaping knowledge, comparison of behaviour, repetition and substitution, antecedents
Bell and Palma 2000 [42]	Not applicable	61	61	NR	NR	NR	—
Erkkola and Makela 1976 [43]	Not stated	103	103	NR	NR	NR	Feedback and monitoring, shaping knowledge, natural consequences
Khaledan et al. 2010 [44]	Not stated	39	—	NR	NR	NR	Goals and planning, feedback and monitoring, repetition and substitution, antecedents
Lee et al. 1996 [45]	Qualified instructor	370	351	NR	NR	NR	Feedback and monitoring, shaping knowledge, comparison of behaviour, repetition and substitution, antecedents
Yeo et al. 2000 [46]	Not stated	17	16	NR	NR	NR	—

Mixed interventions							
Asbee et al. 2009 [47]	Dietician, physician, nurse	144	100	13.02 (5.67)	16.15 (7.03)	*p* = 0.01	Goals and planning, feedback and monitoring, comparison of outcomes, reward and threat
Huang et al. 2011 [48]	Nurse trained in nutrition and fitness	240	189	14.02 (2.38)	16.22 (3.26)	*p* < 0.001	Goals and planning, feedback and monitoring, shaping knowledge, reward and threat
Jeffries et al. 2009 [49]	Medical student	286	236	10.7 (4.21)	11.5 (4.03)	*p* = 0.01 in overweight group but NS in underweight, normal weight or obese	Goals and planning, feedback and monitoring
Phelan et al. 2011 [50]	Research assistants, nurses, clinicians	401	363	Normal weight 16.2 (4.6) obese 15.1 (7.5)	Normal weight 15.3 (4.4) obese 14.7 (6.9)	significant increase in normal weight women exceeding IOM guidelines	Goals and planning, feedback and monitoring, shaping knowledge, associations, antecedents
Polley et al. 2002 [51]	Nutritionist or clinical psychologist	120	110	Normal weight 15.4 (7.1) overweight 13.6 (7.2)	Normal weight 16.4 (4.8); overweight 10.1 (6.2)	significant increase in normal weight women exceeding IOM guidelines	Goals and planning, feedback and monitoring, repetition and substitution
Vinter et al. 2011 [52]	Dietician, physiotherapist	360	304	**median **{**range**} 7.0 (4.7–10.6)	**median **{**range**} 8.6 (5.7–11.5)	*p* = 0.014	Goals and planning, shaping knowledge, comparison of behaviour, repetition and substitution, antecedents
Guelinckx et al. 2010 [53]	Nutritionist	195	122	Active group 9.8 (7.6) Passive group 10.9 (5.6)	10.6 (6.9)	NS	Goals and planning, feedback and monitoring, shaping knowledge, reward and threat
Hui et al. 2011 [54]	Dietician and fitness trainer	52	45	14.2 (5.3)	14.2 (6.3)	NS *p* = 1.00	Goals and planning, feedback and monitoring, shaping knowledge, comparison of behaviour, repetition and substitution, comparison of outcomes, antecedents
Hui et al. 2006 [55]	Dietician and fitness trainer	224	190	15.2 (5.9)	14.1 (6.0)	NS *p* = 0.28	Goals and planning, feedback and monitoring, shaping knowledge, comparison of behaviour, repetition and substitution, comparison of outcomes, antecedents
Jackson et al. 2011 [56]	Video doctor simulating health care provider	321	289	15.15 (NR)	15.24 (NR)	NS *p* = 0.95	—
Bung et al. 1991 [57]	Not stated	41	34	NR	NR	NR	Goals and planning, feedback and monitoring, shaping knowledge, antecedents
Ferrara et al. 2011 [58]	Trained dietician, lactation consultant	197	197	NR	NR	NR	Goals and planning, shaping knowledge, natural consequences, comparison of outcomes
Kulpa et al. 1987 [59]	Nutritionist, exercise physiologist and obstetrician	141	85	Primigravida 14.3 (NR) Multigravida 12.5 (NR)	Prigravida 14.2 (NR) Multigravida 15.4 (NR)	NR	Goals and planning

GWG = gestational weight gain.

SD = standard deviation.

BCT = Behaviour Change Technique.

NR = not reported.

NS = not significant.

IOM = Institute of Medicine.

**Table 2:** Discrepant Behaviour Change Technique categorisation across the studies.

Study	Discrepant BCT categorisation	Type of intervention (D = diet; P = physical activity; M = mixed)
Badrawi et al. 1992 [26]	Goals and planning	D
Barakat et al. 2009 [31]	Goals and planning	P
Barakat et al. 2012 [32]	Goals and planning	P
Bechtel-Blackwell 2002 [22]	Comparison of outcomes	D
Bell and Palma 2000 [42]	Goals and planning	P
Briley et al. 2002 [23]	Goals and planning	D
Baciuk et al. 2008 [29] Cavalcante et al. 2009 [30]	Goals and planning	P
Clapp 1997 [15]	Goals and planning	D
Clapp et al. 2000 [33]	Goals and planning	P
Erkkola 1976 [34]	Goals and planning	P
Erkkola and Makela 1976 [43]	Goals and planning	P
Garshasbi and Faghih 2005 [35]	Goals and planning Shaping knowledge	P
Lee et al. 1996 [45]	Goals and planning	P
Marquez-Sterling et al. 2000 [38]	Goals and planning Shaping knowledge	P
Ney et al. 1982 [18]	Goals and planning	D
Ong et al. 2009 [39]	Goals and planning	P
Prevedel et al. 2003 [40]	Goals and planning	P
Rae et al. 2000 [25]	Goals and planning	D
Santos et al. 2005 [41]	Goals and planning	P
Sedaghati et al. 2007 [28]	Goals and planning	P
Wolff et al. 2008 [21]	Goals and planning	D
Yeo et al. 2000 [46]	Goals and planning Feedback and monitoring Shaping knowledge Repetition and substitution	P

**Table 3:** Prevalence of BCT categories within successful and unsuccessful interventions at reducing gestational weight gain.

	BCTs present in successful intervention (% of 12 studies)	BCTs present in unsuccessful intervention (% of 16 studies)
Goals and planning	75.0	50.0
Feedback and monitoring	91.7	75.0
Shaping knowledge	50.0	81.3
Natural consequences	0	6.3
Comparison of behaviour	8.3	56.3
Associations	8.3	0
Repetition and substitution	16.7	68.8
Comparison of outcomes	25.0	18.8
Reward and threat	16.7	6.3
Regulation	8.3	0
Antecedents	25.0	50.0

BCT = Behaviour Change Technique.
